# Advances in Anticancer Drug Discovery

**DOI:** 10.3390/molecules26071821

**Published:** 2021-03-24

**Authors:** Jóhannes Reynisson

**Affiliations:** School of Pharmacy and Bioengineering, Keele University, Hornbeam Building, Keele, Staffordshire ST5 5BG, UK; j.reynisson@keele.ac.uk; Tel.: +44-(0)1-782-733-985

It has been an absolute pleasure to be the guest editor of this Special Issue! As the title indicates, the topic is on the advances made against cancer. Sixteen manuscripts were published, including eleven research papers and five reviews. The depth and width of the research presented is truly impressive, i.e., the detail of the biological processes within the cancer cells that are elucidated and the diverse nature of the chemical matter under investigation, spanning from heavy metals to small organic molecules.

New synthesized anticancer compounds were introduced in the s-triazine Schiff base derivatives against human breast carcinoma (MCF-7) and colon cancer (HCT-116) cell lines [1], the thioureas chelated with Ru, Os, Rh and Ir as shown in Figure 1 [2], 3-carene derivatives as novel inhibitors of tyrosyl-DNA phosphodiesterase 1 (TDP1), a promising anticancer target [3], and new anthraquinones potentially targeting DNA topoisomerase [4].

The biological mechanism of action was further elucidated for known and new bioactive compounds; 10-gingerol was shown to target lipid rafts in triple negative breast cancer cells [5], a new barbituric acid derivative has antiproliferative and antimigratory effects in hepatocellular carcinoma [6], pyrimethamine, an established antiparasitic drug, was identified in a screen of known drugs against exon 2-depleted splice variant of aminoacyl-tRNA synthetase-interacting multifunctional protein 2, and was effective in mouse xenografts, making it a promising repositioned drug [7], the flavonoid quercetin was tested with docetaxel as a potential adjuvant therapy for breast cancer [8], the immunomodulating drugs thalidomide, lenalidomide, and pomalidomide were tested on cultured normal monocytes, cancer-supporting stromal cells, altering their metabolism and cell–cell communication [9], another flavonoid kushenol Z, isolated from the roots of *Sophora flavescens* used in Traditional Chinese Medicine (TCM), inhibited proliferation and induced apoptosis in non-small-cell lung cancer cells [10], and finally, three new dual inhibitors of tryptophan catabolizing enzymes indoleamine 2,3-dioxygenase 1 (IDO1) and tryptophan 2,3-dioxygenase (TDO2), valid cancer immunotherapy targets, were established using virtual screening of natural products [11].

The review articles were just as varied as the research papers regarding their topics; tannic acid was reviewed for its anticancer activity and its capacity as a drug delivery vehicle [12], the effect of the integrin peptide TNIIIA2 acting as a pro-cancer factor and peptide FNIII14 acting as an anticancer agent was reviewed based on the regulation on β1-integrin activation, which are matricellular proteins existing in association with the extracellular matrix [13], the cytotoxic, anti-inflammatory and anticancer effects of C_17_ and C_18_ acetylenic oxylipins from terrestrial plants were presented and potential mechanisms of action and structural requirements for optimal cytotoxicity were discussed [14], the recent advances in the chemical synthesis and biological evaluation of nucleoside analogues as potential anticancer agents were discussed with the focus on the 4′-heteroatom substitution of the furanose oxygen, 2′-, 3′-, 4′- and 5′-position ring modifications and the development of new prodrug strategies for these compounds, as shown in Figure 2 [15], and the potential of metalloorganic chemistry was reviewed for the mechanism of biological activity of Vanadium complexes, including DNA binding, oxidative stress, cell cycle regulation and programed cell death [16].

In conclusion, an impressive array of new potential therapies is presented in this Special Issue. The number of different approaches to defeat cancer is truly inspiring, giving one hope for the eventual management and cure of most, if not all, cancer types.

## Figures and Tables

**Figure 1:**
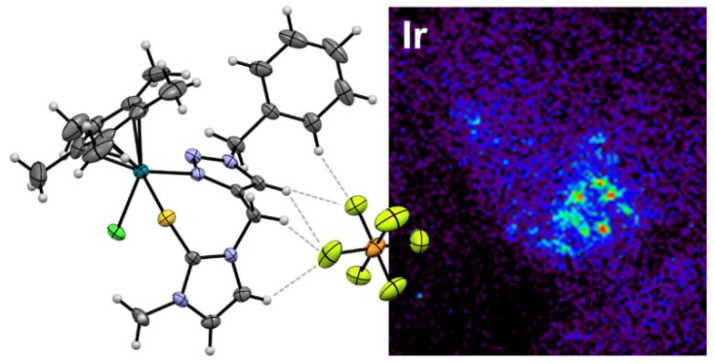
The molecular structure of one of the chelated thiourea enantiomers found (**left**) and Ir distribution in SKOV-3 cancer cells after treatment as determined by X-ray fluorescence microscopy (**right**) [2].

**Figure 2:**
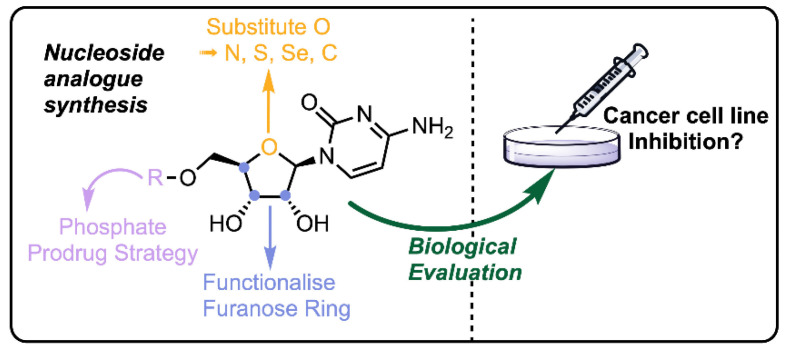
The molecular structures of the reviewed nucleotides [15].
